# Supplementary material for: Multiplexed detection of bacterial nucleic acids using Cas13 in droplet microarrays
Source: PNAS Nexus. 2022 Apr 15;1(1):pgac021. doi: 10.1093/pnasnexus/pgac021 (PMC9013781; doi:10.1093/pnasnexus/pgac021)
Supplement: pgac021_Supplemental_File [file pgac021_supplemental_file.pdf]

Multiplexed detection of bacterial nucleic acids using Cas13 in droplet microarrays

Abstract

Rapid and accurate diagnosis of infections is fundamental to individual patient care and public health management. Nucleic acid detection methods are critical to this effort, but are limited either in the breadth of pathogens targeted or by the expertise and infrastructure required. We present here a high-throughput system that enables rapid identification of bacterial pathogens, bCARMEN, which utilizes: (1) modular CRISPR-Cas13-based nucleic acid detection with enhanced sensitivity and specificity; and (2) a droplet microfluidic system that enables thousands of simultaneous, spatially multiplexed detection reactions at nanoliter volumes; and (3) a novel pre-amplification strategy that further enhances sensitivity and specificity. We demonstrate bCARMEN is capable of detecting and discriminating 52 clinically relevant bacterial species and several key antibiotic resistance genes. We further develop a proof of principle system for use with stabilized reagents and a simple workflow with optical readout using a cell phone camera, opening up the possibility of a rapid point-of-care multiplexed bacterial pathogen identification and antibiotic susceptibility testing.

Supplementary figures:

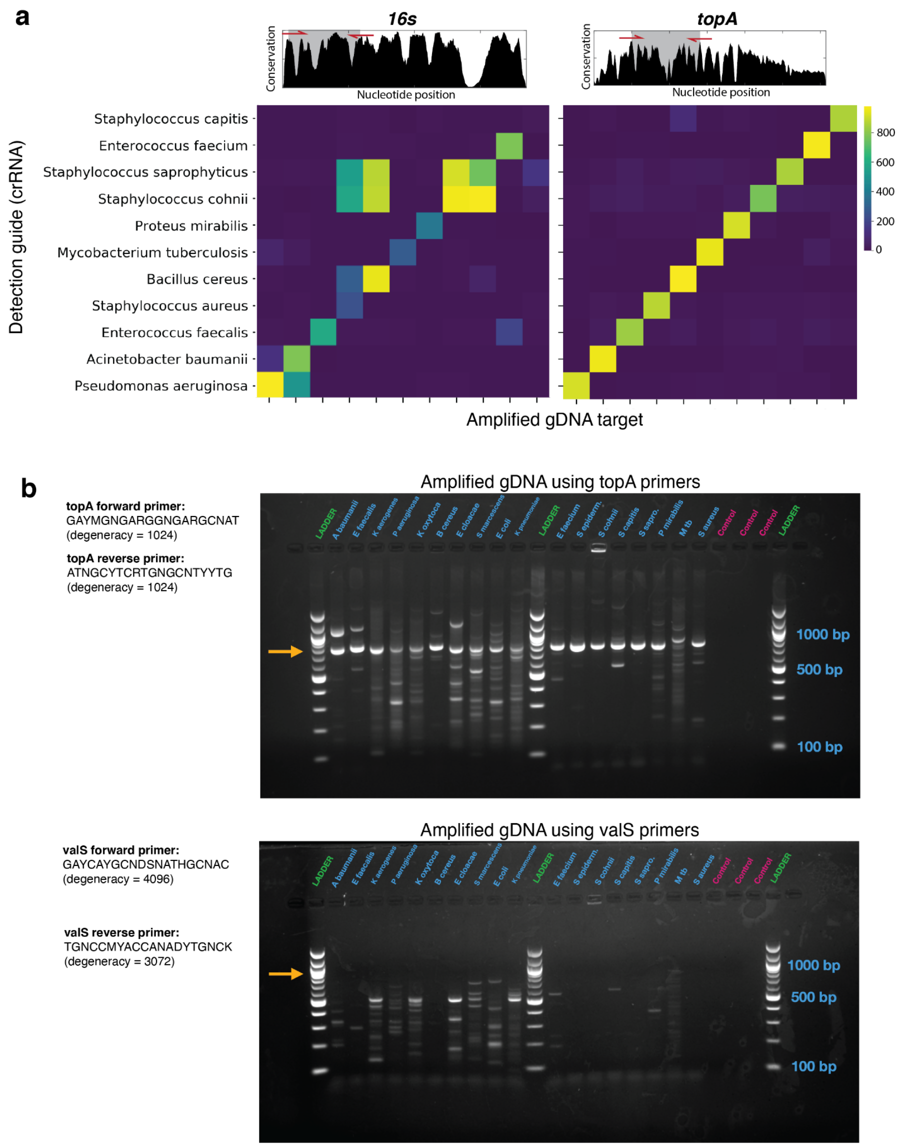

Figure S1: Comparison of performance of different target genes. a, Heatmap showing fluorescent signal from crRNAs targeting a subset of species at 16s vs. topA loci. Genomic loci amplified by primers is depicted above the heatmap. **b**, Agarose gel electrophoresis images of degenerate primer amplification of topA and valS genes across a subset of the 52 bacterial species (expected amplicon length for topA = ~900 bp; valS = ~1000 bp; indicated by yellow arrow)

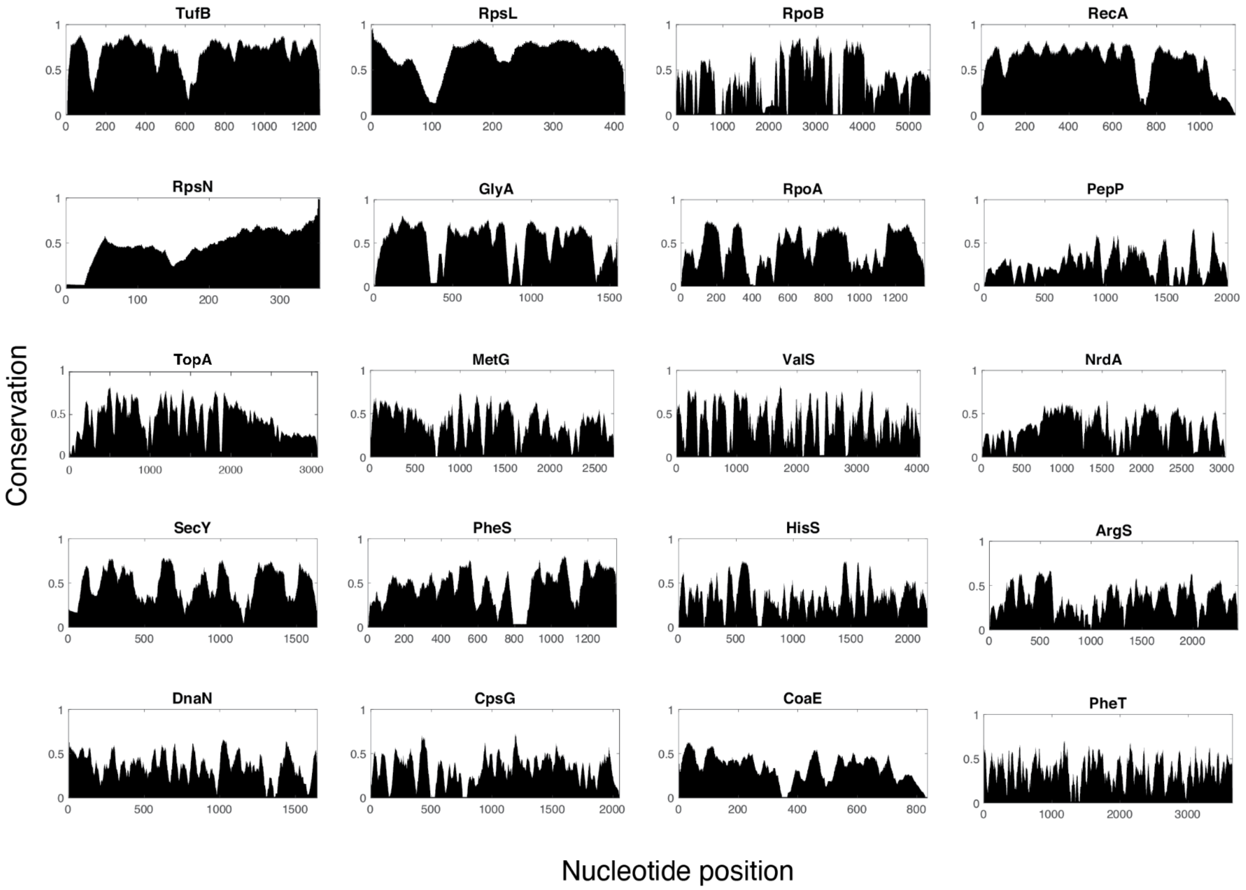

Figure S2: Nucleotide conservation of housekeeping genes across bacterial species
Conservation as a function nucleotide position across 52 bacterial species plotted for 20 housekeeping genes. Conservation score at a given nucleotide position = occurrence of most prevalent nucleotide / 52.

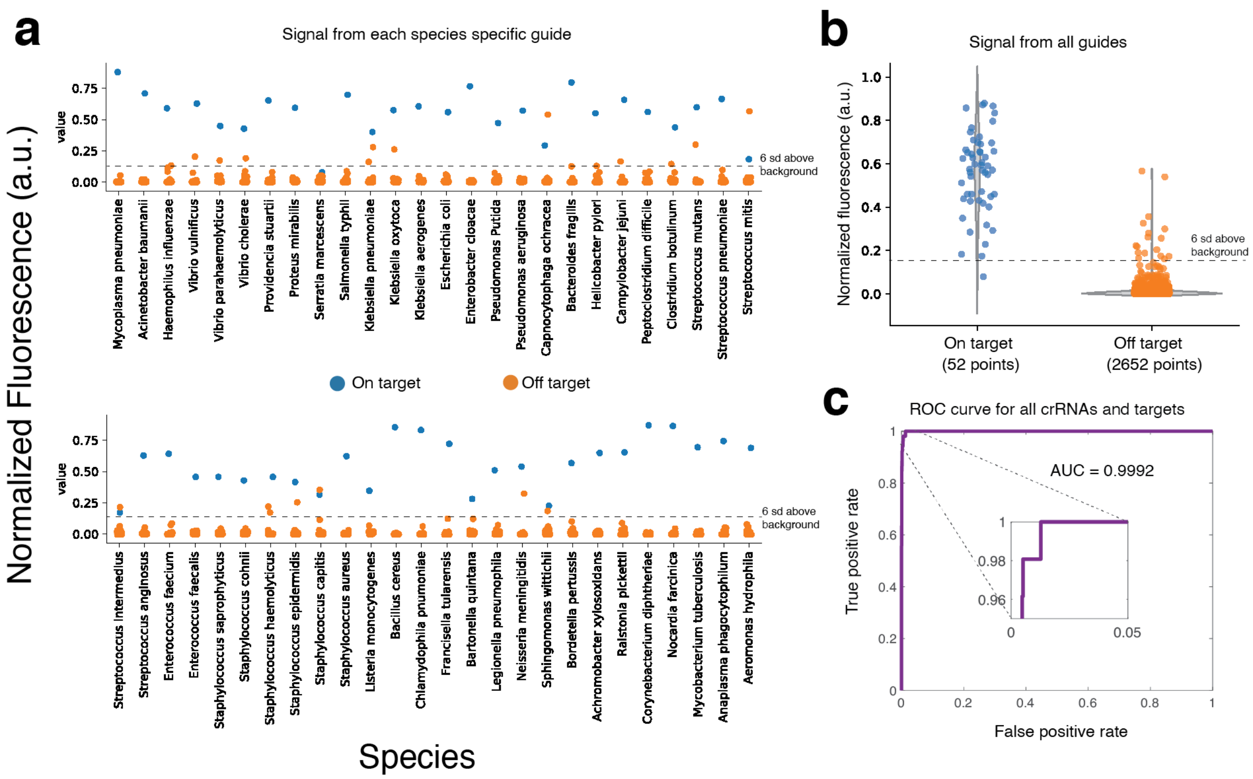

Figure S3: crRNA guide performance in the bacterial panel a, Fluorescent signal for each species-specific crRNA plotted for the target species (blue, on target) and all non-target species (orange, off target.) **b**, Fluorescent signal from all crRNA guides plotted for target species (blue, on target) and all non-target species (orange, off target.) **c**, Receiver operator characteristics curve plotted for on-target vs. off-target signals from all crRNA guides (AUC = 0.9992). (The "6 sd above background" plotted here is based on the mean background across all guides.)

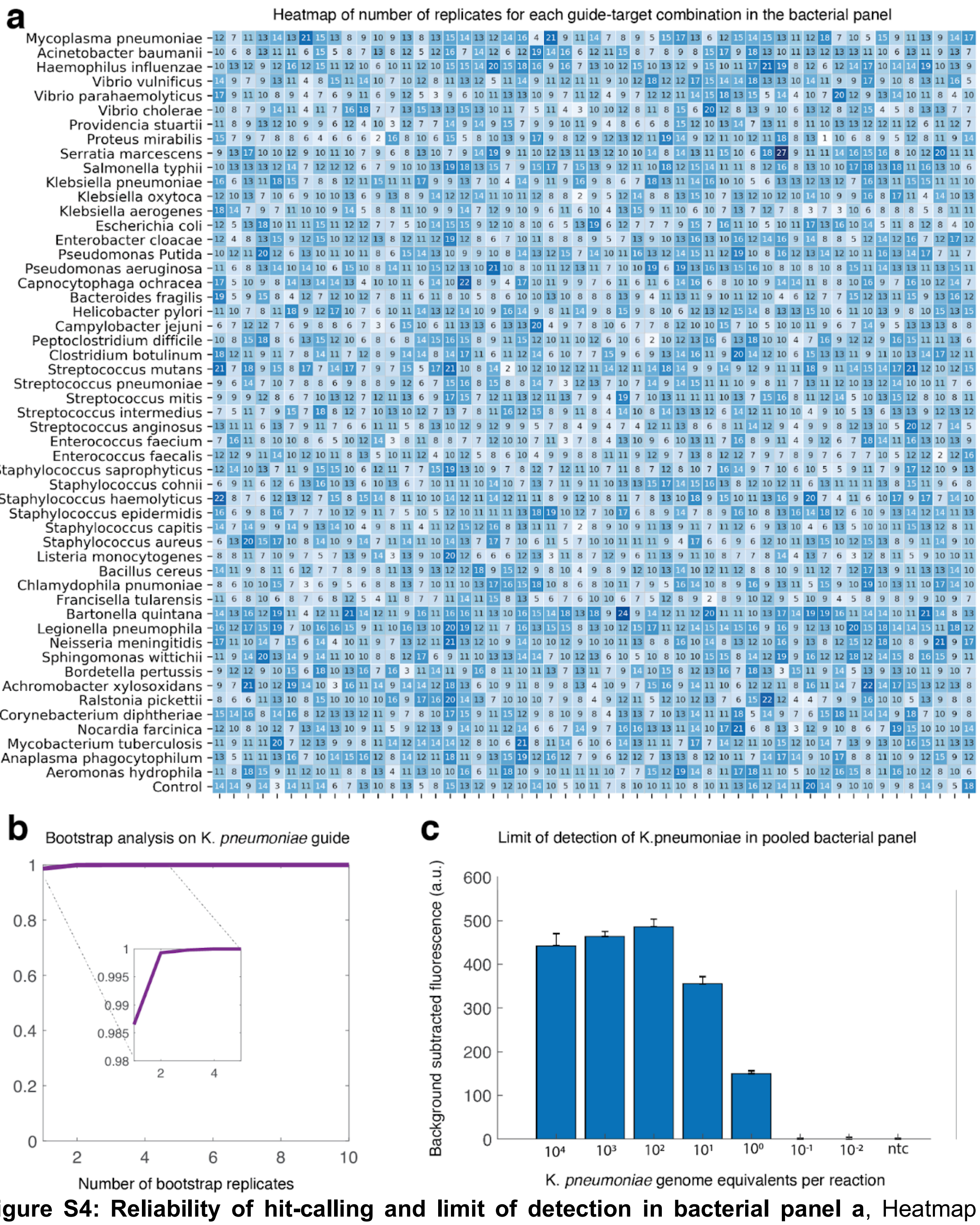

Figure S4: Reliability of hit-calling and limit of detection in bacterial panel a, Heatmap depicting the number of replicates for each crRNA-target pair in the bacterial panel data (Figure 2d) Median = 10.8 **b**, Bootstrap analysis on K. *pneumoniae* guide showing call confidence (fraction of correct calls) as a function of number of replicates. **c**, Limit of detection of the bacterial panel assay

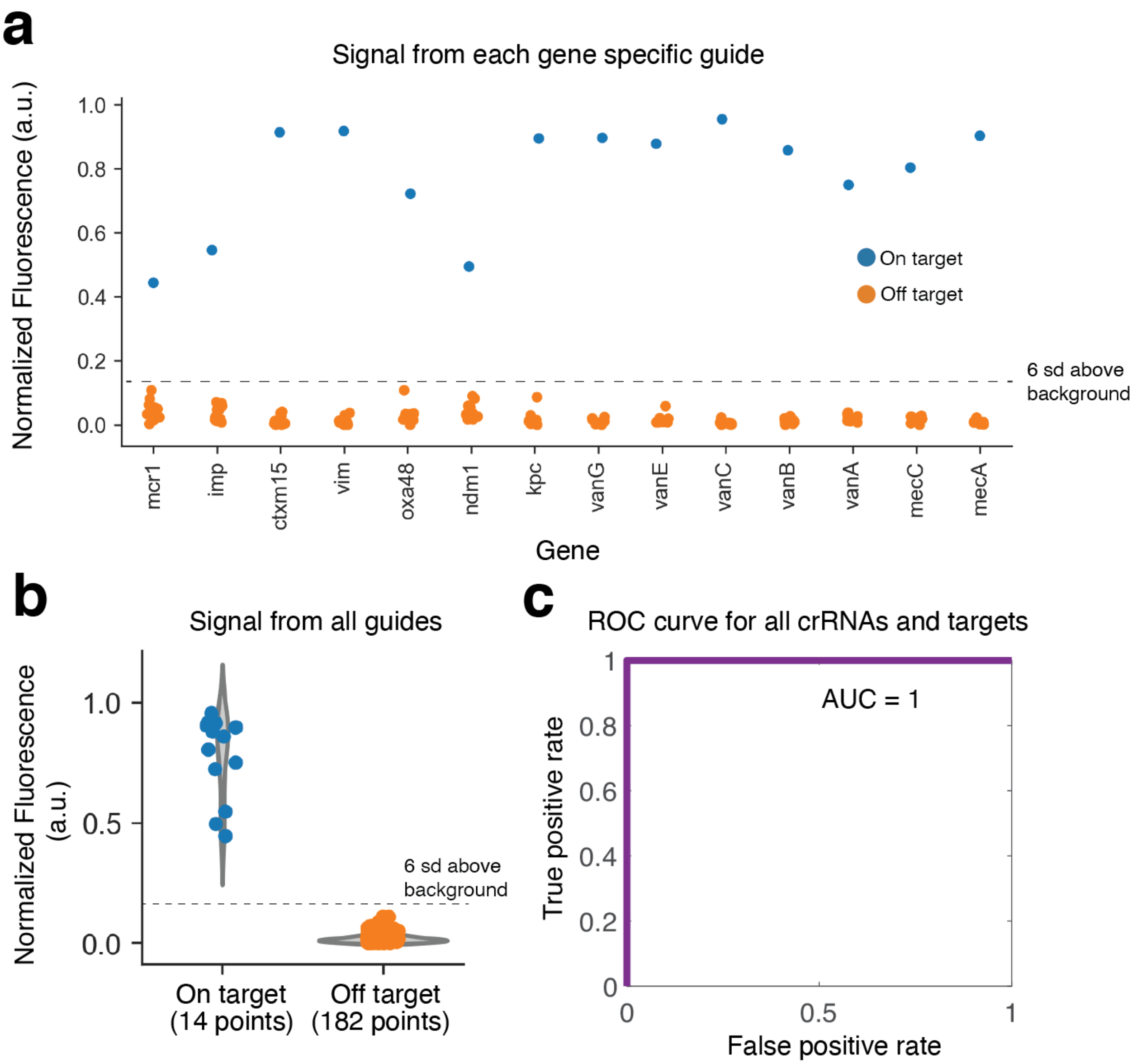

Figure S5: crRNA guide performance in the resistance panel a, Fluorescent signal for each species-specific crRNA plotted for the target gene (blue, on target) and all non-target genes (orange, off target.) **b**, Fluorescent signal from all crRNA guides plotted for target gene (blue, on target) and all non-target genes (orange, off target.) **c**, Receiver operator characteristics curve plotted for on-target vs. off-target signals from all crRNA guides (AUC = 1). (The “6 sd above background” plotted here is based on the mean background across all guides.)

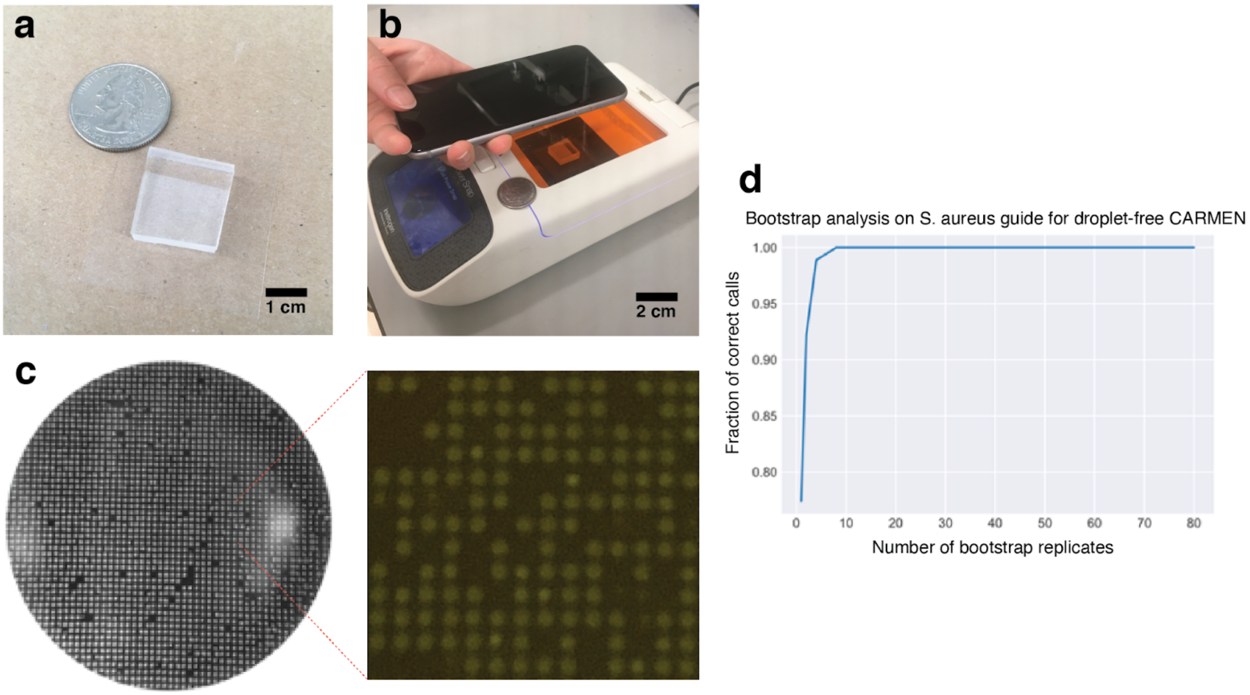

Figure S6: Droplet-free assay setup and bootstrap analysis a, Freeze-dried chip loaded with target mix on PCR film with an American quarter dollar coin shown for scale. **b**, Imaging setup used for droplet-free assay. **c**, Cell phone image of microwells with a zoomed in image showing the droplets close up. d, Bootstrap analysis on S. aureus topA guide plotting the fraction of correct calls as a function of number of replicates.

Table S1: Strain information for bacterial species panel			
**Species**	**Tested strain name**	**Source**	**Number of strains used in guide design**
Mycoplasma pneumoniae	DSM 23978	DSMZ	4
Acinetobacter baumanii	RB197	BWH clinical isolate	14
Haemophilus influenzae	DSM4690	DSMZ	20
Vibrio vulnificus	DSM10143	DSMZ	5
Vibrio parahaemolyticus	RIMD 2210633	Goldberg lab (MGH)	12
Vibrio cholerae		MGH clinical isolate	98
Providencia stuartii	DSM4539	DSMZ	2
Proteus mirabilis	RB037	BWH clinical isolate	5
Serratia marcescens	BWH_23	BWH clinical isolate	10
Salmonella typhii	NR-514	BEI	45
Klebsiella pneumoniae	RB011	BWH clinical isolate	15
Klebsiella oxytoca	RB078	BWH clinical isolate	10
Klebsiella aerogenes	RB251	MGH clinical isolate	3
Escherichia coli	RB001	MGH clinical isolate	53
Enterobacter cloacae	RB250	MGH clinical isolate	15
Pseudomonas putida	WCS358	Lory lab	10
Pseudomonas aeruginosa	RB019	BWH clinical isolate	16
Capnocytophaga ochracea	DSM7271	DSMZ	2
Bacteroides fragilis	DSM2151	DSMZ	3
Helicobacter pylori	CPY6081	BEI	51
Campylobacter jejuni	NR-3057	BEI	14
Peptoclostridium difficile	CD104	BEI	4
Clostridium botulinum	NR-2713	BEI	13
Streptococcus mutans	DSM20523	DSMZ	10
Streptococcus pneumoniae	D39	Lipsitch lab	24
Streptococcus mitis	Spar10	BEI	27
Streptococcus intermedius	F0413	BEI	3
Streptococcus anginosus	F0211	BEI	8
Enterococcus faecium	RB029	BWH clinical isolate	4
Enterococcus faecalis	RB027	BWH clinical isolate	5
Staphylococcus saprophyticus	RB497	Wadsworth lab clinical isolate	2
Staphylococcus cohnii	RB493	Wadsworth lab clinical isolate	3
Staphylococcus haemolyticus	DNF00585	BEI	3
Staphylococcus epidermidis	RB494	Wadsworth lab clinical isolate	8

Staphylococcus capitis	RB492	Wadsworth lab clinical isolate	3
Staphylococcus aureus	Newman	BEI	40
Listeria monocytogenes	10403s	Goldberg lab (MGH)	29
Bacillus cereus	TOR16585	BEI	37
Chlamydophila pnumoniae	DSM19748	DSMZ	5
Francisella tularensis	NR-3015	BEI	10
Bartonella quintana	JK39	BEI	3
Legionella pneumophila	Lp01 JK40	Isberg lab (Tufts)	29
Neisseria meningitidis	NM3222	BEI	12
Sphingomonas wittichii	DSM6014	DSMZ	1
Bordetella pertussis	H921	BEI	3
Achromobacter xylosoxidans	DSM2042	DSMZ	2
Ralstonia pickettii	DSM6297	DSMZ	3
Corynebacterium diphtheriae	DSM44123	DSMZ	11
Nocardia farcinica	DSM43665	DSMZ	1
Mycobacterium tuberculosis	H37Rv	Lab	21
Anaplasma phagocytophilum	NR-51150	BEI	20
Aeromonas hydrophila	DSM30187	DSMZ	2

Table S2: Primer and guide sequences for bacterial species panel

Species	Forward Primer Sequence	Reverse Primer Sequence	Guide (reverse complement with T7 promoter attached)
Mycoplasma pneumoniae	gaaatTAATACGACTCACTATAGGGAGACCGTGAGGGAGAGGCAATTTCCTGACA	AGTTGGGTGAATTGCTTCATGGGCGTCTTG	AAGGACAAGTCGAAGTGCCAACGCATTAGTTTTAGTCCCCTTCGTTTTTGGGGTAGTCTAAATCCCCTATAGTGAGTCGTATTAatttc
Acinetobacter baumanii	gaaatTAATACGACTCACTATAGGGGGATAGAGAAGGGGAAGCAATCGCTTGGCA	TTGATGGACGAATAGCTTCGTGGGCTTCTTG	CAGCCAACACGTCTCGACTTAAACCGTGGTTTTAGTCCCCTTCGTTTTTGGGGTAGTCTAAATCCCCTATAGTGAGTCGTATTAatttc
Haemophilus influenzae	gaaatTAATACGACTCACTATAGGGGATAGAGAAGGGGAAGCGATTGCGTGGCA	TGATGGACGAATAGCCTCATGAGCCTCTTG	ACGATGATCGTTTTAGTCGCGTGGTGTTGTTTTAGTCCCCTTCGTTTTTGGGGTAGTCTAAATCCCCTATAGTGAGTCGTATTAatttc
Vibrio vulnificus	gaaatTAATACGACTCACTATAGGGACCGCGAGGGGGAAGCTATTGCGTGGCA	TGAAGGACGAATCGCTTCGTGTGCTTCTTG	AGCTATTGCGTGGCACCTTCGTGAGATCGTTTTAGTCCCCTTCGTTTTTGGGGTAGTCTAAATCCCCTATAGTGAGTCGTATTAatttc
Vibrio parahaemolyticus	gaaatTAATACGACTCACTATAGGGACCGCGAGGGGGAAGCAATCGCATGGCA	AGATGGACGAATCGCTTCGTGCGCTTCTTG	CGATGAAGAGCGATACAAACGAGTAGTCGTTTTAGTCCCCTTCGTTTTTGGGGTAGTCTAAATCCCCTATAGTGAGTCGTATTAatttc
Vibrio cholerae	gaaatTAATACGACTCACTATAGGGACCGCGAGGGGGAAGCAATCGCATGGCA	AGATGGACGAATCGCTTCGTGCGCTTCTTG	CGATGAAAAGCGATATAAACGAGTGGTAGTTTTAGTCCCCTTCGTTTTTGGGGTAGTCTAAATCCCCTATAGTGAGTCGTATTAatttc
Providencia stuartii	gaaatTAATACGACTCACTATAGGGGACCGCGAAGGGGAAGCTATTGCATGGCA	GAAGGACGGATAGCTTCGTGCGCTTCTTG	CATGGATCGCGTAGTAGGTTATATGGTAGTTTTAGTCCCCTTCGTTTTTGGGGTAGTCTAAATCCCCTATAGTGAGTCGTATTAatttc
Proteus mirabilis	gaaatTAATACGACTCACTATAGGGACCGCGAAGGAGAAGCTATCGCGTGGCA	AGAAGGACGAATAGCTTCGTGCGCTTCTTG	ATGCCATTACACAAGCATTCCAAACACCGTTTTAGTCCCCTTCGTTTTTGGGGTAGTCTAAATCCCCTATAGTGAGTCGTATTAatttc
Serratia marcescens	gaaatTAATACGACTCACTATAGGGACCGCGAAGGGGAAGCCATTGCCTGGCA	AAGGACGAATCGCCTCGTGCGCTTCCTG	CGATCTGCTGGCGAAGGGTGAAACCGCGGTTTTAGTCCCCTTCGTTTTTGGGGTAGTCTAAATCCCCTATAGTGAGTCGTATTAatttc
Salmonella typhii	gaaatTAATACGACTCACTATAGGGACCGCGAAGGGGAAGCCATTGCATGGCA	AAGGGCGGATCGCTTCGTGCGCTTCCTG	TTGTCGGCAGGTCGCGTTCAGTCTGTCGGTTTTAGTCCCCTTCGTTTTTGGGGTAGTCTAAATCCCCTATAGTGAGTCGTATTAatttc
Klebsiella pneumoniae	gaaatTAATACGACTCACTATAGGGACCGCGAAGGGGAAGCCATTGCCTGGCA	GAAGGACGAATCGCTTCGTGCGCTTCCTG	CAGCCGTGATGAGACCATGGCGGCGGTAGTTTTAGTCCCCTTCGTTTTTGGGGTAGTCTAAATCCCCTATAGTGAGTCGTATTAatttc
Klebsiella oxytoca	gaaatTAATACGACTCACTATAGGGACCGCGAAGGGGAAGCCATCGCATGGCA	ATGGACGAATCGCCTCGTGCGCTTCCTG	GTCACGCATAACGGCGATAAGCCGTTCCGTTTTAGTCCCCTTCGTTTTTGGGGTAGTCTAAATCCCCTATAGTGAGTCGTATTAatttc
Klebsiella aerogenes	gaaatTAATACGACTCACTATAGGGACCGCGAAGGGGAAGCCATTGCCTGGCA	GAAGGACGAATCGCTTCGTGCGCTTCCTG	TATCGGTGGTGATGAGCAACGGTACAGCGTTTTAGTCCCCTTCGTTTTTGGGGTAGTCTAAATCCCCTATAGTGAGTCGTATTAatttc
Escherichia coli	gaaatTAATACGACTCACTATAGGGACCGCGAAGGGGAAGCCATTGCATGGCA	GAAGGACGAATCGCTTCGTGCGCTTCCTG	ATTGGTGGTGATGATGCGCGCTATAGCCGTTTTAGTCCCCTTCGTTTTTGGGGTAGTCTAAATCCCCTATAGTGAGTCGTATTAatttc
Enterobacter cloacae	gaaatTAATACGACTCACTATAGGGACCGCGAAGGGGAAGCCATTGCCTGGCA	GAAGGACGAATGGCTTCGTGCGCTTCCTG	AAAGAATGCGATTCGTCAGGCGTTTGAGGTTTTAGTCCCCTTCGTTTTTGGGGTAGTCTAAATCCCCTATAGTGAGTCGTATTAatttc
Pseudomonas putida	gaaatTAATACGACTCACTATAGGGACCGCGAAGGGGAAGCCATTGCCTGGCA	AAGGCCGAATCGCTTCGTGCGCCTCCTG	GGCACCGCCAAGAACGCCAAGGTGCGTTGTTTTAGTCCCCTTCGTTTTTGGGGTAGTCTAAATCCCCTATAGTGAGTCGTATTAatttc
Pseudomonas aeruginosa	gaaatTAATACGACTCACTATAGGGCGCGAAGGGGAGGCCATCGCCTGGCA	AGGACGGATCGCCTCGTGCGCTTCCTG	TTCTCCCAGCCCGGCGAGCTCGATATCAGTTTTAGTCCCCTTCGTTTTTGGGGTAGTCTAAATCCCCTATAGTGAGTCGTATTAatttc
Capnocytophaga ochracea	gaaatTAATACGACTCACTATAGGGAAGACCGTGAAGGAGAAGCTATTGCGTTTCA	GTAGGACGAATGGCTTCGTGCGCCTCTTG	ACTCTCAGCAGGACGCGTTCAGTCGGTAGTTTTAGTCCCCTTCGTTTTTGGGGTAGTCTAAATCCCCTATAGTGAGTCGTATTAatttc
Bacteroides fragilis	gaaatTAATACGACTCACTATAGGGGACCGGGAGGGAGAAGCCATTTCATGGCA	TCGGGCGGATAGCTTCGTGCGCTTCCTG	GCAGGCACGCCGTATCTTAGACCGTATCGTTTTAGTCCCCTTCGTTTTTGGGGTAGTCTAAATCCCCTATAGTGAGTCGTATTAatttc
Helicobacter pylori	gaaatTAATACGACTCACTATAGGGAGACAGAGAGGGGGAAGCGATAGGCTATCA	GTGGGCCTGATCGCTTCATGGGCTTCTTG	TTTCATGAGATCACGCAAAATGCGATTTGTTTTAGTCCCCTTCGTTTTTGGGGTAGTCTAAATCCCCTATAGTGAGTCGTATTAatttc
Campylobacter jejuni	gaaatTAATACGACTCACTATAGGGAGGATAGAGAAGGAGAGGCTATAGCCTATCA	TTGTAGGGCGTATAGCTTCATGGGCTTCTTG	GCGTGTTCAAAGTGCGGCTTTAAAGATCGTTTTAGTCCCCTTCGTTTTTGGGGTAGTCTAAATCCCCTATAGTGAGTCGTATTAatttc
Peptoclostridium difficile	gaaatTAATACGACTCACTATAGGGCCTGATAGAGAAGGTGAAGCTATATCATGGCA	AGGTTGGTCTTATTGCCTCATGGGCATCTTG	ACAGGCAAGAAGAGTGCTAGACAGACTTGTTTTAGTCCCCTTCGTTTTTGGGGTAGTCTAAATCCCCTATAGTGAGTCGTATTAatttc
Clostridium botulinum	gaaatTAATACGACTCACTATAGGGCGGATAGAGAGGGAGAAGCTATTTCTTGGCA	TGGTTGGTCTTATAGCTTCATGGGCATCCTG	AGATTAGTTGGATATAAAATAAGCCCTAGTTTTAGTCCCCTTCGTTTTTGGGGTAGTCTAAATCCCCTATAGTGAGTCGTATTAatttc
Streptococcus mutans	gaaatTAATACGACTCACTATAGGGGGACCGTGAAGGAGAAGCGATTTCTTGGCA	AGACGGACGAATAGCCTCATGAGCATCCTG	CGTAATGCACCGCTTCCTTATACGACATGTTTTAGTCCCCTTCGTTTTTGGGGTAGTCTAAATCCCCTATAGTGAGTCGTATTAatttc
Streptococcus pneumoniae	gaaatTAATACGACTCACTATAGGGGGACCGTGAAGGAGAAGCGATTTCTTGGCA	GACGGACGAATAGCCTCATGGGCATCCTG	GTTCAGTCCATTGCCCTTAAACTCATCAGTTTTAGTCCCCTTCGTTTTTGGGGTAGTCTAAATCCCCTATAGTGAGTCGTATTAatttc
Streptococcus mitis	gaaatTAATACGACTCACTATAGGGGGACCGTGAAGGAGAAGCGATTTCTTGGCA	AGACGGACGAATAGCCTCATGAGCATCCTG	CGCGTTCAGTCTATTGCGCTTAAGTTAAGTTTTAGTCCCCTTCGTTTTTGGGGTAGTCTAAATCCCCTATAGTGAGTCGTATTAatttc
Streptococcus intermedius	gaaatTAATACGACTCACTATAGGGCAGATCGAGAAGGAGAAGCTATTTCTTGGCA	TCGATGGACGAATCGCTTCATGAGCATCTTG	AGAAGGGGTTATCGGCTGGACGTGTACAGTTTTAGTCCCCTTCGTTTTTGGGGTAGTCTAAATCCCCTATAGTGAGTCGTATTAatttc
Streptococcus anginosus	gaaatTAATACGACTCACTATAGGGGGACCGTGAAGGAGAAGCTATTTCTTGGCA	GACGGACGAATCGCTTCATGGGCGTCTTG	TAAAGAACCGCGCCAGATTGACATGGATGTTTTAGTCCCCTTCGTTTTTGGGGTAGTCTAAATCCCCTATAGTGAGTCGTATTAatttc
Enterococcus faecium	gaaatTAATACGACTCACTATAGGGGGATAGAGAAGGAGAAGCGATTGCTTGGCA	GTTGGACGCACAGCTTCATGGGCATCTTG	AGTGCAGGACGTGTCCAATCTGTCGCTTGTTTTAGTCCCCTTCGTTTTTGGGGTAGTCTAAATCCCCTATAGTGAGTCGTATTAatttc
Enterococcus faecalis	gaaatTAATACGACTCACTATAGGGGGACCGAGAAGGTGAAGCAATTGCTTGGCA	GACGGACGAACCGCTTCATGGGCATCTTG	CGTTTAGTTGGATACTCGATTAGTCCTAGTTTTAGTCCCCTTCGTTTTTGGGGTAGTCTAAATCCCCTATAGTGAGTCGTATTAatttc
Staphylococcus saprophyticus	gaaatTAATACGACTCACTATAGGGCGACCGTGAAGGTGAAGCTATTGCTTGGCA	AGACGGTCTGATGGCTTCATGGGCATCTTG	ACAAGCACGACGTATTCTAGATCGTTTAGTTTTAGTCCCCTTCGTTTTTGGGGTAGTCTAAATCCCCTATAGTGAGTCGTATTAatttc
Staphylococcus cohnii	gaaatTAATACGACTCACTATAGGGCGATCGTGAAGGTGAGGCAATTGCTTGGCA	TAGATGGTCTAATAGCTTCGTGGGCATCTTG	GTGGATGCACAACAGGCTAGAAGAATTTGTTTTAGTCCCCTTCGTTTTTGGGGTAGTCTAAATCCCCTATAGTGAGTCGTATTAatttc
Staphylococcus haemolyticus	gaaatTAATACGACTCACTATAGGGCGACCGTGAAGGTGAAGCAATTGCTTGGCA	TTGAAGGTCTAATAGCCTCATGGGCATCTTG	GCTGTCTGCAGGGCGAGTTCAATCTGTAGTTTTAGTCCCCTTCGTTTTTGGGGTAGTCTAAATCCCCTATAGTGAGTCGTATTAatttc
Staphylococcus epidermidis	gaaatTAATACGACTCACTATAGGGTGACCGTGAAGGTGAAGCGATTGCTTGGCA	CTAGTAGGTCTAATAGCTTCGTGAGCATCTTG	GCTGGGAGAGTTCAGTCAGTTGCTTTACGTTTTAGTCCCCTTCGTTTTTGGGGTAGTCTAAATCCCCTATAGTGAGTCGTATTAatttc
Staphylococcus capitis	gaaatTAATACGACTCACTATAGGGGACCGTGAAGGTGAAGCGATTGCCTGGCA	TTGTAGGTCTAATCGCTTCATGGGCATCTTG	GTTCAGTCAGTAGCTCTTCGTTTAGTTAGTTTTAGTCCCCTTCGTTTTTGGGGTAGTCTAAATCCCCTATAGTGAGTCGTATTAatttc
Staphylococcus aureus	gaaatTAATACGACTCACTATAGGGCGACCGTGAAGGTGAAGCAATTGCTTGGCA	TTGAAGGTCTAATAGCCTCATGGGCATCTTG	AGAGCTTGAAGATTCTAAAGAAAATCGCGTTTTAGTCCCCTTCGTTTTTGGGGTAGTCTAAATCCCCTATAGTGAGTCGTATTAatttc
Listeria monocytogenes	gaaatTAATACGACTCACTATAGGGAGACCGCGAAGGAGAAGCAATTGCATGGCA	TTGTAGGTCTGATTGCTTCATGGGCATCTTG	TAGAGTTAGATCAATCAGACAAACTACGGTTTTAGTCCCCTTCGTTTTTGGGGTAGTCTAAATCCCCTATAGTGAGTCGTATTAatttc
Bacillus cereus	gaaatTAATACGACTCACTATAGGGAGACCGCGAAGGAGAAGCTATTGCTTGGCA	AAGTAGGACGAATTGCCTCATGCGCATCTTG	CAAGCAAGACGTATACTAGATCGTCTTGGTTTTAGTCCCCTTCGTTTTTGGGGTAGTCTAAATCCCCTATAGTGAGTCGTATTAatttc
Chlamydophila pnumoniae	gaaatTAATACGACTCACTATAGGGTGATAGAGAAGGAGAGGCAATTGCCTGGCA	AGTGGGACGTATGGCTTCGTGAGCATCTTG	CTGGCACATCGCGAATCAGCTTCCTGACGTTTTAGTCCCCTTCGTTTTTGGGGTAGTCTAAATCCCCTATAGTGAGTCGTATTAatttc
Francisella tularensis	gaaatTAATACGACTCACTATAGGGCCAGATAGAGAAGGTGAAGCTATATCATGGCA	TGTGACACGGATAGCTTCGTGTGCTTCTTG	ATCTATCGTGTCACATTTAACGAAATCAGTTTTAGTCCCCTTCGTTTTTGGGGTAGTCTAAATCCCCTATAGTGAGTCGTATTAatttc
Bartonella quintana	gaaatTAATACGACTCACTATAGGGCTGATCGTGAAGGGGAAGCTATTTCATGGCA	TTGGCCGGATGGCTTCATGCGCTTCCTG	TATCGATACATCACTTGTAGATGCCTACGTTTTAGTCCCCTTCGTTTTTGGGGTAGTCTAAATCCCCTATAGTGAGTCGTATTAatttc

Legionella pneumophila	gaaatTAATACGACTCACTATAGGGTGATAG<br>AGAGGGAGAAGCTATCTCGTGGCA	AAGTTGGCCTAATGGCTTCATGT<br>GCTTCCTG	ATCGGCCGGACGAGTACAAAGCCCTGCCGTTTTAGTCCCCTTCGT<br>TTTTGGGGTAGTCTAAATCCCCTATAGTGAGTCGTATTAatttc
Neisseria meningitidis	gaaatTAATACGACTCACTATAGGGGATAGG<br>GAAGGCGAAGCCATTTCCTGGCA	TCGGACGGATGGCTTCGTGCGCT<br>TCTTG	AGGATGCTGTGCGCAAACTCGGCTTCACGTTTTAGTCCCCTTCGT<br>TTTTGGGGTAGTCTAAATCCCCTATAGTGAGTCGTATTAatttc
Sphingomonas wittichii	gaaatTAATACGACTCACTATAGGGCGCGAG<br>GGGGAGGCGATCAGCTGGCA	CGGGCGGATCGCCTCATGCGCTT<br>CCTG	TCACCTTCAACGCGATCACCAAGGCCGCGTTTTAGTCCCCTTCGT<br>TTTTGGGGTAGTCTAAATCCCCTATAGTGAGTCGTATTAatttc
Bordetella pertussis	gaaatTAATACGACTCACTATAGGGGGCGCG<br>AAGGCGAACTGATCTTCCGCTA	TGTTGTCGAAGATGCGCCGGTTG<br>GGCTTG	GCAGACGCCCACGCTGGCCATCGTCAATGTTTTAGTCCCCTTCGT<br>TTTTGGGGTAGTCTAAATCCCCTATAGTGAGTCGTATTAatttc
Achromobacter xylosoxidans	gaaatTAATACGACTCACTATAGGGGGCGCG<br>AGGGCGAACTGATCTTCCGCTA	TGTTGTCGAAGATGCGGCGGTTC<br>GGCTTG	AGCGCGAGGAACGCATCCGCCGCTTCGTGTTTTAGTCCCCTTCGT<br>TTTTGGGGTAGTCTAAATCCCCTATAGTGAGTCGTATTAatttc
Ralstonia pickettii	gaaatTAATACGACTCACTATAGGGGGCGCG<br>AAGGCGAGTTGATCTTCCGCCT	CTGTTGTCGAAGATCCGCTTGTTC<br>GGCTTG	GATCGTGGTGGAGCGCGAAGAGAAGATCGTTTTAGTCCCCTTCGT<br>TTTTGGGGTAGTCTAAATCCCCTATAGTGAGTCGTATTAatttc
Corynebacterium diphtheriae	gaaatTAATACGACTCACTATAGGGACCGCG<br>AGGGTGAAGCCATCGCTTGGCA	CTGGACGGATTGCCTCGTGGGCT<br>TCTTG	CTACGGCTACGAGGTATCCCCAGTGCTGGTTTTAGTCCCCTTCGT<br>TTTTGGGGTAGTCTAAATCCCCTATAGTGAGTCGTATTAatttc
Nocardia farcinica	gaaatTAATACGACTCACTATAGGGCGCGAG<br>GGCGAGGCCATCGCCTGGCA	CGGACGGATCGCCTCGTGTGCCT<br>CCTG	ACTCGACCCGGACCTGGTCGACGCGCAGGTTTTAGTCCCCTTCGT<br>TTTTGGGGTAGTCTAAATCCCCTATAGTGAGTCGTATTAatttc
Mycobacterium tuberculosis	gaaatTAATACGACTCACTATAGGGACCGTG<br>AGGGCGAAGCTATTGCCTGGCA	GGGCCGGATAGCCTCGTGCGCTT<br>CCTG	CCTCAAACCGCGCATACCGGTAAAGCGGGTTTTAGTCCCCTTCGT<br>TTTTGGGGTAGTCTAAATCCCCTATAGTGAGTCGTATTAatttc
Anaplasma phagocytophilum	gaaatTAATACGACTCACTATAGGGGATCGC<br>GAAGGAGAGGCGATAGCTTGGCA	GTTGGACGGATAGCTTCGTGCGC<br>TTCCTG	AGATGTTACAGTGAACAGGATGGTGTTCGTTTTAGTCCCCTTCGTT<br>TTTGGGGTAGTCTAAATCCCCTATAGTGAGTCGTATTAatttc
Aeromonas hydrophila	gaaatTAATACGACTCACTATAGGGGATAGA<br>GAGGGAGAAGCGATCGCCTGGCA	GGGCGGATCGCCTCGTGGGCCT<br>CCTG	CCAAGACCGCCATCCAGGAGGCGTTTGCGTTTTAGTCCCCTTCGT<br>TTTTGGGGTAGTCTAAATCCCCTATAGTGAGTCGTATTAatttc

Table S3: Stain information for resistance panel and CARMEN v2		
**Strain label**	**Species**	**Source**
MSSA	Staphylococcus aureus	Reference strain
MRSA1	Staphylococcus aureus	BWH clinical isolate
MRSA2	Staphylococcus aureus	Barczak lab at MGH
CSE1	Klebsiella pneumoniae	BWH clinical isolate
CSE2	Klebsiella pneumoniae	BWH clinical isolate
CRE1	Klebsiella pneumoniae	BIDMC clinical isolate
CRE2	Klebsiella pneumoniae	
CRE3	Klebsiella pneumoniae	
CRE4	Klebsiella pneumoniae	
CRE5	Klebsiella pneumoniae	
CRE6	Escherichia coli	
CRE7	Escherichia coli	MGH clinical isolate
CRE8	Klebsiella pneumoniae	BWH clinical isolate
CRE9	Escherichia coli	ATCC
CRE10	Klebsiella pneumoniae	ATCC
CRE11	Klebsiella pneumoniae	Wadsworth lab clinical isolate
CRE12	Klebsiella pneumoniae	Wadsworth lab clinical isolate
CRE13	Escherichia coli	Wadsworth lab clinical isolate
CRE14	Escherichia coli	Wadsworth lab clinical isolate
CRE15	Escherichia coli	Wadsworth lab clinical isolate
CRE16	Klebsiella pneumoniae	MGH clinical isolate
CRE17	Klebsiella pneumoniae	
VSE	Enterococcus faecalis	Barczak lab at MGH
VRE1	Enterococcus faecium	Barczak lab at MGH
VRE2	Enterococcus faecalis	BWH clinical isolate
VRE3	Enterococcus faecalis	BWH clinical isolate
MSSA	Staphylococcus aureus	Reference strain
MRSA	Staphylococcus aureus	BWH clinical isolate

Table S4: Primer and guide sequences for resistance panel

Gene	Forward primer	Reverse primer	Guide (reverse complement with T7 promoter attached)
mecA	gaaatTAATACGACTCACTATAGGGTCAACAAGTTCCAGATTACAACTTCACCAG	ATCTGATGATTCTATTGCTTGTTTTAAGTCGATA	GCAATGATTGGGTTAAATAACAAAACATGTTTTAGTCCCCTTCGTTTTTGGGGTAGTCTAAATCCCCTATAGTGAGTCGTATTAatttc
mecC	gaaatTAATACGACTCACTATAGGGTCAACAAATTTCAAATCACTACATCACCAG	GTCTGATGATTCTATTGCTTGCTTTAAATCGATA	TCTATTATAGCCTTAAAAGAAAATAAACGTTTTAGTCCCCTTCGTTTTTGGGGTAGTCTAAATCCCCTATAGTGAGTCGTATTAatttc
vanA	gaaatTAATACGACTCACTATAGGGTACATTGGAATTACGAAATCTGGTGTATGG	ACTTGCCATGCAAAGCTGAAAATGCTACAT	GCACGGATTACTTGTTAAAAAGAACCATGTTTTAGTCCCCTTCGTTTTTGGGGTAGTCTAAATCCCCTATAGTGAGTCGTATTAatttc
vanB	gaaatTAATACGACTCACTATAGGGTACATCGGAATTACAAAAAACGGTGTATGG	ATTTGCCATGCAAAACCGGGAAAGCCACAT	GCATGGGCTGCTTGTCATGAAAGAAAGCGTTTTAGTCCCCTTCGTTTTTGGGGTAGTCTAAATCCCCTATAGTGAGTCGTATTAatttc
vanC	gaaatTAATACGACTCACTATAGGGACCATTGGCATCGCACCAACAATGGATTGG	ACTTCCCATGCAAGACTGGAAAGAGGACAT	TTCTAGCCAAGGATTTATATTAGGAGAAGTTTTAGTCCCCTTCGTTTTTGGGGTAGTCTAAATCCCCTATAGTGAGTCGTATTAatttc
vanE	gaaatTAATACGACTCACTATAGGGAAAATAGGGATCACCGAAGAAGGTCATTGG	AACCTCCATGTAAAACTGGGAATAAAATAT	CTGTGAAGAAATCGTAGTTGATTTCGCAGTTTTAGTCCCCTTCGTTTTTGGGGTAGTCTAAATCCCCTATAGTGAGTCGTATTAatttc
vanG	gaaatTAATACGACTCACTATAGGGCCAATAGGAATTACAAGAAGTGGTGAATGG	TTTTGCCATGCAATACGGGGAATACCAAAT	ATCTATGCCCTGTTGTCGTTTCCCAAAAGTTTTAGTCCCCTTCGTTTTTGGGGTAGTCTAAATCCCCTATAGTGAGTCGTATTAatttc
kpc	gaaatTAATACGACTCACTATAGGGCGTCTAGTTCTGCTGTCTTGTCTCTCATGG	CAAAGTCCTGTTCGAGTTTAGCGAATGGTT	GCTGGCTGGCTTTTCTGCCACCGCGCTGGTTTTAGTCCCCTTCGTTTTTGGGGTAGTCTAAATCCCCTATAGTGAGTCGTATTAatttc
ndm1	gaaatTAATACGACTCACTATAGGGAATGTCTGGCAGCACACTTCCTATCTCGAC	TGATCTCCTGCTTGATCCAGTTGAGGATCT	CAACGGTTTGATCGTCAGGGATGGCGGCGTTTTAGTCCCCTTCGTTTTTGGGGTAGTCTAAATCCCCTATAGTGAGTCGTATTAatttc
oxa	gaaatTAATACGACTCACTATAGGGTTAAAATTCCCAATAGCTTGATCGCCCTCG	ATAAACAGGCACAACTGAATATTTCATCGC	CCAAGTCTTTAAGTGGGATGGACAGACGGTTTTAGTCCCCTTCGTTTTTGGGGTAGTCTAAATCCCCTATAGTGAGTCGTATTAatttc
vim	gaaatTAATACGACTCACTATAGGGGATGAGTTGCTTYTKATTGATACAGCKTGG	GATAGAARASYTCTACKGGACCGAARCGCA	CTCGCGGAGATTGAAAAGCAAATTGGACGTTTTAGTCCCCTTCGTTTTTGGGGTAGTCTAAATCCCCTATAGTGAGTCGTATTAatttc
imp	gaaatTAATACGACTCACTATAGGGAMAGATACTGAAAADTTAGTHAVTTGGTTT	CAGGYARCCAAACYACTASRTTATCTKGAG	GAATAGAGTGGCTTAATTCTCAATCTATGTTTTAGTCCCCTTCGTTTTTGGGGTAGTCTAAATCCCCTATAGTGAGTCGTATTAatttc
ctxm15	gaaatTAATACGACTCACTATAGGGATAAAACCGGCAGCGGTGGCTATGG	GCTAATACATCGCGACGGCTTTCTGCCTTA	ATCGTGCGCCGCTGATTCTGGTCACTTAGTTTTAGTCCCCTTCGTTTTTGGGGTAGTCTAAATCCCCTATAGTGAGTCGTATTAatttc
mcr-1	gaaatTAATACGACTCACTATAGGGGCTCGTTGGCTTAGATGACTTTGTCGCTGC	GCTTAAAATACGCAGGCCCGTGATTGCCCA	GGCAAAGATATGCTGATCATGCTGCACCGTTTTAGTCCCCTTCGTTTTTGGGGTAGTCTAAATCCCCTATAGTGAGTCGTATTAatttc
